# ‘Multiple-test’ approach to the laboratory diagnosis of tuberculosis -perception of medical doctors from Ujjain, India

**DOI:** 10.1186/s12879-015-1037-2

**Published:** 2015-08-11

**Authors:** Manju Raj Purohit, Megha Sharma, Senia Rosales-Klintz, Cecilia Stålsby Lundborg

**Affiliations:** Department of Pathology, R. D. Gardi Medical College, Ujjain, India; Central Clinical Laboratory, Ujjain Charitable Trust Hospital and Research Centre, Ujjain, India; Global Health (IHCAR), Department of Public Health Sciences, Karolinska Institutet, Stockholm, Sweden; Department of Pharmacology, R.D. Gardi Medical College, Ujjain, India

**Keywords:** Tuberculosis, Diagnosis, Challenges

## Abstract

**Background:**

Delay in diagnosis is one of the most important factors for the control of tuberculosis (TB) in endemic countries like India. As laboratory diagnosis is the mainstay for identification of active disease, we aim to explore and understand the opinions of medical doctors about the laboratory diagnosis of TB in Ujjain, India.

**Methods:**

Sixteen qualified specialist medical doctors from Ujjain were purposefully selected for the study. Individual interviews with the doctors (13 men and 3 women), were conducted. As one interview could not be completed, data from15 interviews were analyzed using manifest and latent content analysis.

**Results:**

Based on perception of the doctors, the theme; ‘challenges and need for the laboratory diagnosis of TB’ emerged from the following subthemes: (i) Relationship between basic element of the TB diseases process such as ‘Symptoms prior to diagnoses’ and ‘Clinical characteristics of TB’, which were not specific enough to diagnose TB (ii) The prevailing conditions such as lack of explicit diagnostic tools, lead to the doctors using the ‘multiple tests’ or ‘empiric treatment’ approach (iii) The doctors proposed that there is a need for access to a rapid, single and simple diagnostic test, and a need for awareness and knowledge of the practitioners regarding specific TB investigations, and early referral to improve the situation at resource-limited settings.

**Conclusion:**

The medical specialists use a ‘multiple test’ or ‘empiric treatment’ approach to diagnose TB. According to the participants, there is a low dependence and uptake of the available laboratory TB investigations by medical practitioners. There is an urgent need to have a specific, simple and reliable test, and a protocol, to improve diagnosis of TB and to prevent development of resistant TB.

## Background

In 1993, the World Health Organization (WHO) declared tuberculosis (TB) a global health emergency and launched direct observed treatment short course (DOTS) to control it. The cornerstone of DOTS strategy is rapid and accurate diagnosis of all forms of TB. The disease, however, remains one of the most intractable health challenges in low- and middle-income countries. Estimates from 2013 showed that about 56 % of all cases in world are in the South-East Asia and Western Pacific Regions collectively. India carries 24 % of the global total TB cases, which is highest among TB endemic countries [1].

Research has highlighted several challenges in attaining the objectives of the DOTS process such as limited knowledge about signs and symptoms of TB, and delays in obtaining diagnoses and in receiving treatment [2–6]. Even with subsidized TB diagnostic services in endemic countries, only half of the sputum smear–positive cases are detected [7]. In India, TB diagnosis practices vary and the major possible reasons for this might be poor public health care infrastructure, a vast private health care sector and informal health care providers that are not regulated by the national TB program [8]. It has been noticed that providers prefer to diagnose TB only based on chest X-ray findings or signs and symptoms rather than by doing sputum smear examinations [9]. A study from India indicates that TB patients are inadequately investigated and TB investigations are not a priority before treatment by the providers [7]. The diagnostic delays are therefore mainly related to the health system [10]. This results in a four-fold delay in the diagnosis of TB after the patient’s first contact with the health system, which leads to poor disease prognosis and increased transmission of the disease in the community [11, 12].

The WHO acknowledged diagnostic delays in TB and realized that to a large extent the available TB laboratory capacity is insufficient to address the diagnostic challenges. Consequently, the WHO is now giving priority to strengthening laboratory services and uptake of new evidence-based tuberculosis diagnostic approaches into routine practice in endemic countries. As confirmation of TB is mainly a laboratory-dependent procedure, it is important to understand the function of the laboratory in the diagnostic process of TB, and various practice related factors influencing it in the local health system. There is a lack of studies describing and assessing factors and needs affecting the acceptability and implementation of new laboratory diagnostics tools. This study therefore looks at the laboratory diagnosis of TB from the perspective of medical doctors to explore their real life experience, the existing situation, and the requirements of laboratory diagnosis of TB, in Ujjain district, India.

## Methods

### Study setting

**Fig. 1:**
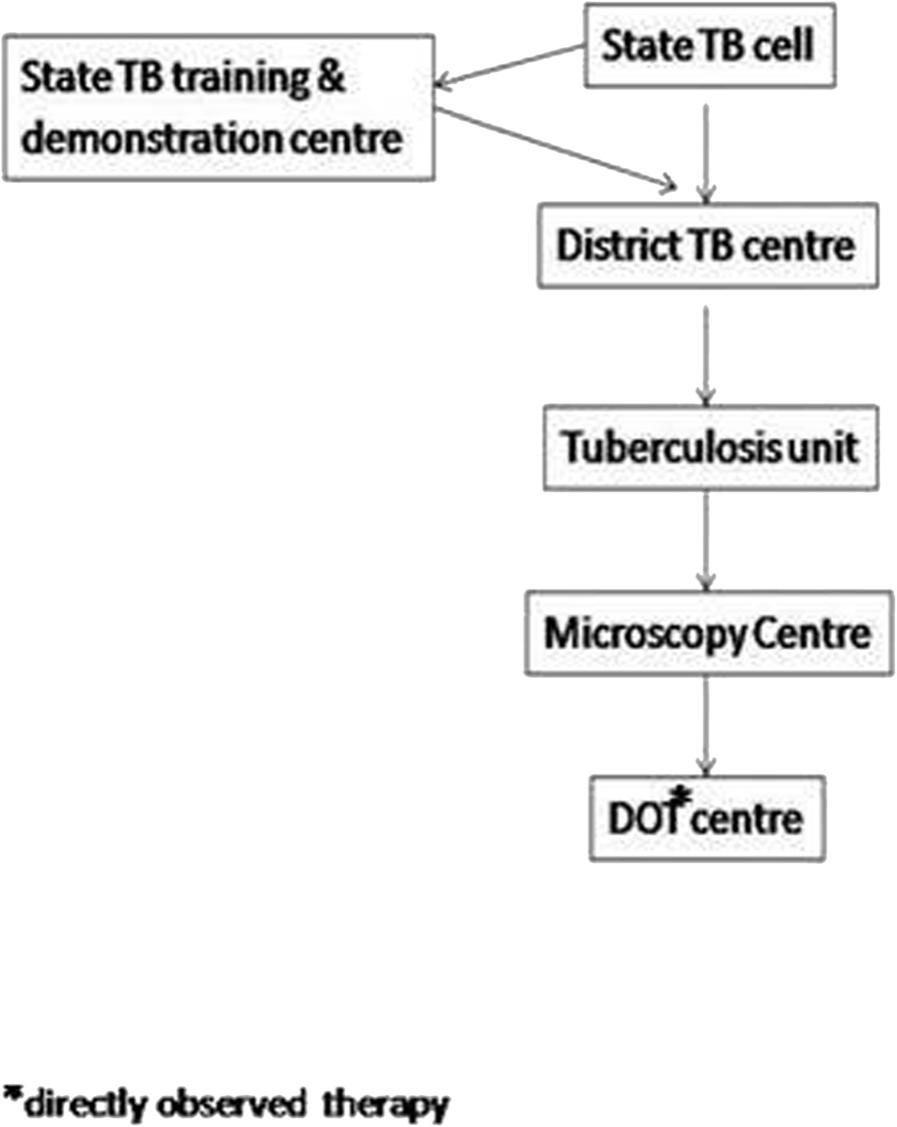
Structure of RNTCP at state level (adapted from RNCTP guideline, 2011)

The study was conducted in Ujjain, central India, between April and August 2013. The population of Ujjain district is 1.98 million [13], of which 60 % live in rural areas. The Revised National Tuberculosis Control Program (RNTCP) was implemented in 2003 Ujjain. It provides a vertical top-down approach with the district tuberculosis centre as the central point for performing diagnosis, treatment, logistics and reporting. The treatment unit’s area link between district level and peripheral health care centers, non-government organizations, and private sector microscopy centers (Fig. 1). The district has large and heterogeneous private sector healthcare facilities, including untrained providers and formally trained doctors, though the trained doctors are mainly concentrated in urban areas. The private sector provides TB care to a high number of patients. Most of the medical expenses in the private sector for many illnesses are paid for out-of-pocket by the patients as medical insurance is limited [7].

### Participants and data collection instruments

Individual interviews were conducted with 16 medical doctors (13 men and 3 women). Inclusion criteria for the participants was that they have to be qualified and specialized doctors from -different specialties, with more than 10 years of medical experience and should have experience with tuberculosis patients. Doctors employed in both the public and private sector in Ujjain (private sector defined as one where user fees are a source of income) were included in the study (Table 1). From over 50 qualified and specialized doctors practicing in Ujjain city, 21 doctors were eligible according to the inclusion criteria. Seventeen doctors from different major specialties were approached, and 16 were interviewed. Most (4/5) of the selected doctors from public sector were also treating patients in their private clinics. The interview guide was developed based on discussions with peers and co-authors who have experience in various fields of public health and laboratory work in resource-limited settings. The interview guide consisted of semi-structured, open-ended introductory probing questions regarding (i) clinical history and findings for TB (ii) general and specific laboratory investigations for TB diagnosis (iii) experience and awareness of laboratory and diagnostic modalities.

**Table 1:** Participants’ characteristics of study conducted among medical doctors in Ujjain, Central India for the laboratory diagnosis of tuberculosis

Participant code	Age range (in years)	Specialty	Type of health sector	Work experience (in years)
P1	40-45	Specialist physician	Private	15
P2	40-45	Specialist physician	Private	13
P3	60-65	Specialist physician	Public and private	36
P4	60-65	General physician	Public and private	37
P5	30-35	General physician	Public and private	11
P6	35-40	Pediatrician	Private	12
P7	35-40	Pediatrician	Private	10
P8	55-60	ENT surgeon	Private	27
P9	40-45	ENT surgeon	Private	13
P10	50-55	Gynecologist	Private	26
P11	45-50	Gynecologist	Private	20
P12	40-45	Lung and chest diseases specialist	Private	15
P13	55-60	Surgeon	Private	30
P14	55-60	Orthopedic surgeon	Public and private	24
P15	55-60	Surgeon	Public	23

### Data collection

The interviews were conducted by the first author (MRP), who is fluent in both English and Hindi, in locations chosen by the participants, mostly in participants’ clinics in the absence of patients and clinic staff. On an average, an interview lasted for 40–50 min. The interviews were audio-recorded after getting verbal consent from the participants. The interviews were carried out in a mix of Hindi (local language) and English as per the convenience of participants. The audio files were transcribed in English by the first author or by an assistant on the same day or as soon as possible. The data collection continued until data saturation was achieved.

### Analysis

Of the sixteen interviews, one interview could not be completed, as the interviewee had to attend an emergency and it was not possible to appoint a suitable time to finish the interview later. This partially conducted interview was not included in the analysis. The interview transcripts were analyzed by manifest and latent content analysis [14]. The analysis consisted of identifying meaning units, condensing it into condensed units and assigning codes to identical condensed units. Open codes were generated and organized manually, and similar codes were grouped into tentative categories. Categories were then organized, leading to the identification of sub-themes and a theme. The data was regularly discussed with one of the co-authors (MS) for underlying subthemes and themes. The categories were discussed with all the co-authors until a consensus was reached.

### Ethics

The Ethics Committee of R.D. Gardi Medical College approved the study (number 187/2012). The participants gave verbal informed consent for voluntary participation in the study and audio recording of the interviews. Participants were assured of the confidentiality regarding the content and their identification.

## Results

The theme, ‘Challenges and need for the laboratory diagnosis of TB’ emerged from the three subthemes; (i) Relationship between basic elements of TB and the diagnostic process such as ‘Symptoms prior to diagnoses’ and ‘Clinical characteristics of TB’ which were not specific enough to diagnose TB (ii) Contextual and prevailing conditions for TB diagnosis as lack of explicit and reliable diagnostic tools and consumerism led the doctors to use the ‘multiple-tests’ or the ‘empiric-treatment’ approach to diagnose TB and extra-pulmonary TB (iii) The proposed context-relevant need for interventions for TB diagnosis by the participants: the need and accessibility of a rapid, single and simple diagnostic test; awareness and knowledge of the practitioners regarding specific TB investigations; and early referral to improve the situation at resource-limited settings.

**Fig. 2:**
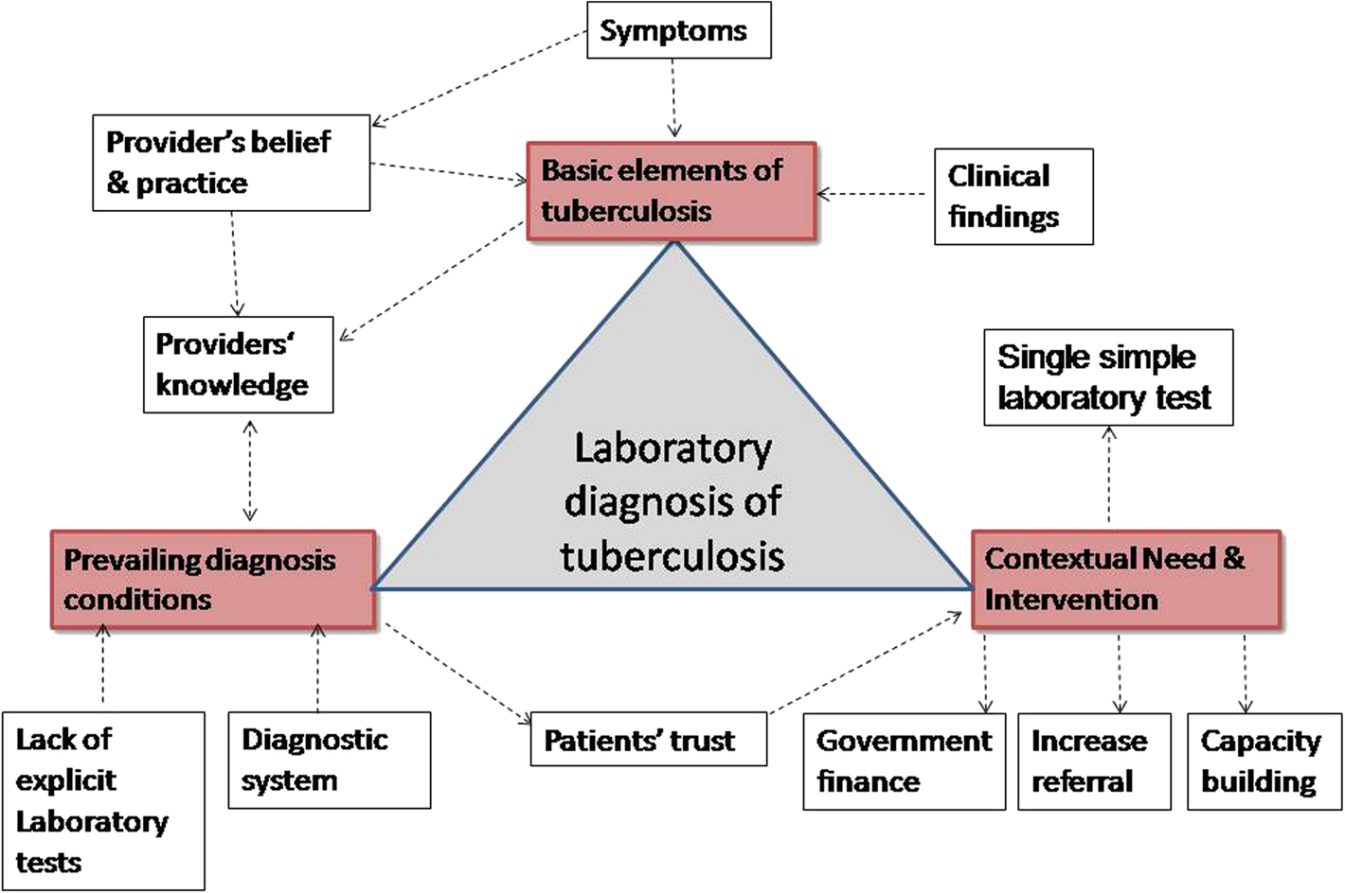
Schematic representation of dynamic of laboratory diagnosis of tuberculosis based on the findings of the study conducted among medical doctors at Ujjain, Central India

Table 2 provides the coding framework of domains of challenges and need of laboratory diagnosis. In the following text sub-themes, categories and codes are described with some illustrative quotations from the interviewees in italics. Explanations by the authors are presented in square brackets. The dynamic of laboratory diagnosis of TB that were identified from the transcripts is depicted in Fig. 2.

**Table 2:** Domains of challenges and need for the laboratory diagnosis of tuberculosis from study conducted among medical doctors in Ujjain, Central India

Theme	Sub-theme	Categories	Codes
Challenges and need for the laboratory diagnosis of TB^a^	*Relationship between the basic elements of TB and the diagnostic process*	Symptoms prior to diagnosis	Cough, Low-grade fever, on –specific symptoms
		Clinical characteristics of TB	Non-specific clinical signs, importance of clinical examination, many clinical signs together need to be interpreted
		Inter-relationship between beliefs, knowledge and practice	TB stigma associated with socioeconomic status, traditional beliefs, Utilization of traditional health services, TB related illiteracy
	*Contextual and prevailing conditions for TB diagnosis*	Laboratory system in action	Improper knowledge of laboratory test, low knowledge of newer TB test, poor adherence to protocol, self made protocols, non-availability of TB specific tests, multiple tests prescription, empiric treatment is considered as diagnosis test, wrong notion for the TB tests
		Lack of explicit and reliable diagnostic tool	No definitive laboratory tests, laboratory test are considered supportive, multiple test result help diagnosis, sputum for AFB^b^ is unreliable, PCR^c^ and culture not useful in routine practice
		Consumerism and the diagnostic process	Private practice, choice of tests, diagnostic algorithm
		Extra-pulmonary tuberculosis and the diagnostic process	Importance of clinical history, Importance of clinical examination, difficult laboratory diagnosis, use of various modalities, invasive procedure
	*Context relevant need and intervention*	Organizational changes and education of health care providers and improving referral	Early referral, easy accessibility, availability of infrastructure, education, use of common modes of communication
		Formulation of simple and effective diagnostic protocol for all kind of TB	Simple test, reliable diagnostic tool, guidelines

^a^tuberculosis; ^b^acid-fast-bacilli; ^c^polymerase chain reaction

### Sub-theme 1: Relationship between the basic elements of TB and the diagnostic process

Certain factors appeared as the ‘Basic elements’, a set of conditions pertaining to TB disease process that challenges its diagnosis. The subtheme emerged from the following categories: (i) Symptoms prior to diagnosis (ii) Clinical characteristics of TB (iii) Inter-relationship between beliefs, knowledge, and practice of TB diagnosis.

#### Category 1.1: Symptoms prior to diagnosis

All participants were aware of the various symptoms of TB. History of fever, cough and loss of weight were considered important factors for TB diagnosis. Many participants emphasized that symptoms are important but not specific for diagnosis of TB as stated in the following quote:

*Clinical history is important to make diagnosis but it is not specific, [long pause] actually no single symptom is specific of TB …P11*

#### Category 1.2: Clinical characteristics of TB

According to most participants, a proper detailed systemic examination is important to observe signs related to tuberculosis. All participants believed that different clinical signs are non–specific to suspect TB in a patient. However, they insist on investigations for TB when multiple clinical characteristic are present in a particular patient.

*I have not seen any case where a specific general appearance of patients helps me to diagnose TB [short pause] it is not very specific to give a clue to the diagnosis of TB [short pause]single sign may not or cannot point out TB but all the clinical findings when taken together are important to suspect TB …P5*

#### Category 1.3: Inter-relationship between beliefs, knowledge, and practice of TB diagnosis

We discovered that strategies taken for diagnosing TB by a doctor were influenced by their beliefs and knowledge about tuberculosis. Respondents considered a cough to be normal for smokers and as a part of cold. Patients with long duration of fever (of 2–3 weeks) were also not considered for investigating sputum for acid-fast bacilli(AFB). According to some respondents, village doctors (both non-specialized qualified doctors posted at primary health centers and non-qualified persons practicing in villages) or *‘Jhola-chhap’* (Hindi) also called ‘small doctors’ (non-qualified health care providers)treat patients for fever or cough until the patient gets some complication of TB or the general condition deteriorated severely. They believed that the patients from rural areas usually develop late stage of the disease before they present to the health services. Some participants assumed that only a specialist doctor should order sputum examination for AFB and other TB-specific investigations. According to respondents, patients have distrust in investigations that are performed at the primary health centre:

*…most of the city patients don’t visit civil hospital especially high socioeconomic class patients never visit there......according to them it is against their status to avail government services while poor -class patients feel the services are not of good quality.....the report they get are wrong and of poor quality.....* P13

*…most of the patients from periphery take a long treatment before reaching or referred to me from ‘small doctors’ doctors so it is late for diagnosis and miss treated ......it is late phase of disease ....by the time patient comes he develops deformity which is avoidable …*P12

### Sub-theme 2: *Contextual and prevailing conditions for TB diagnosis*

Contextual and prevailing conditions are circumstances that act or interact on patients during the process of laboratory diagnosis of TB in the local conditions. The subtheme emerged from the following categories: (i) Laboratory system use for confirmation of TB (ii) Lack of explicit and reliable diagnostic tools (iii) Consumerism and the diagnostic process (iv) Extra-pulmonary tuberculosis (EPTB) and the diagnostic process.

#### Category 2.1: Laboratory tests for confirmation of TB

All participants knew the importance of laboratory investigations for diagnosis of TB but complained that no single laboratory test is sensitive or specific enough to consider as a stand-alone method for confirmation of TB. Patients were investigated simultaneously with many laboratory tests. According to the participants, a ‘multiple-tests’ approach helps them to reach an early diagnosis by having positive result in at least one of the investigations. There was poor adherence of the doctors to the recommended algorithm for investigating a patient suspected to have TB. They stated that some investigations would also help in monitoring the patient during treatment. Sputum examination for AFB and Mantoux tests was considered TB-specific investigations. Some participants also considered erythrocyte sedimentation rate (ESR) and serum antibody assays as TB-specific tests, but most participants doubted the reliability of ESR for TB diagnosis work-up. Culture and polymerase chain reaction (PCR) for *Mycobacterium tuberculosis* were not considered helpful for the diagnosis of TB in routine clinical settings, as these tests were not available at all health facilities. PCR was, however, preferred over taking sputum cultures to confirm TB. Most of the doctors considered ‘response to empiric treatment of TB’ as the ‘*diagnostic procedure*’ for confirmation of TB as evident in the following quote:

*…All these tests may not show a direct evidence of TB but together give a clue and then a course of empiric or supportive anti-Koch’s treatment is started without laboratory diagnosis [short pause] to see the response of treatment for confirmation of TB …P8*

#### Category 2.2: lack of explicit and reliable diagnostic tools

All laboratory investigations for TB were believed to be non-specific by most participants. The results of all TB laboratory tests, whether as simple as ESR and Mantoux test, or as complicated as PCR and culture, were considered insufficient to confirm the diagnosis. Participants judged that a panel of laboratory tests carries more clinical value than a single test. They also had the opinion that sometimes a combination of diagnostic modalities such as radio-imaging, laparoscopy and biopsy are important to finalize the diagnosis.

*…no single test is good enough to give final diagnosis and [short pause], I get indirect evidence from other supportive investigations such as X-ray, CT [computed tomography] scan [short pause] MRI [magnetic resonance imaging] or by invasive procedures as laparoscopy findings-or biopsy or aspiration cytology …P4*

#### Category 2.3: Consumerism and the diagnostic process

We noticed that doctors tried to combat their fear of losing a paying customer (patient) by fulfilling patients’ expectation of quick relief. Instead of disclosing suspicion of TB and advising TB investigations, practitioners started the patient on empiric treatment. Meeting the preferences of patients was also illustrated during tests election, as requesting PCR was associated with the stigma of TB. The socioeconomic status of the patients influenced respondents in the TB investigation process. Participants emphasized the need to have evidence for TB before initiation of anti-tubercular therapy for high socioeconomic status and literate patients. The fear of legal litigation by a literate and high socioeconomic status patient promoted doctors to request laboratory investigations for them. With low socioeconomic patients, empiric treatment is more often ordered than in affluent patients, as doctors believed that it helps to save money of poor patients, as is reflected in this quote:

*…In poor patients the response to treatment is better for diagnosis [short pause] as patients are satisfied if doctor didn’t prescribed many tests and patients feel that the doctor has treated me well in few days without spending lot of money on investigations and waiting for reports. P1*

#### Category 2.4: Extra-pulmonary tuberculosis (EPTB) and the diagnostic process

All participants understood that EPTB disease is suspected only when local signs and symptoms of a particular EPTB site developed in a patient. They found that the confirmation of a laboratory diagnosis of EPTB is much more difficult than of pulmonary TB. The EPTB diagnosis may require invasive procedures or supportive evidence from other modalities. Practitioners had a common opinion that health personnel at public health facilities have poor training in diagnosing EPTB, which leads to poor referral to specialized services as is indicated in the quote:

*…clinical examination is very important to reach the diagnosis especially for the kind of TB, I come across as TB of neck, where clinical findings lead me to investigate and to include or exclude the possibility of diagnosis of tuberculosis. Village doctors usually keep on treating these patients symptomatically for long until some complications develops, [short pause] they don’t consider TB for neck swelling …P9*

### Sub-theme 3: Proposed context-relevant needs for interventions for TB diagnosis

Participants brought up problems with the current test procedures and suggested a few context relevant solutions. The subtheme emerged from the following categories: (i) Education of health care providers and improving referral (ii) Formulation of simple and effective diagnostic protocols for all kinds of TB.

#### Category 3.1: Organizational changes and education of healthcare providers and improving referral

The ‘non-qualified practitioners’ seen as ‘doctors’ and village doctors should have specific training on the TB program including early referral of suspected TB cases for sputum microscopy. They felt that the rates of referral should be improved by providing incentives to healthcare workers. Village doctors should also have awareness of recent diagnostics and knowledge of TB symptomatology, especially of extra-pulmonary or atypical forms of the disease. Functioning equipment such as X-ray machines and microscopes should be available at all township level healthcare facilities. General community awareness of TB should also be raised using different modes of communication.

*…I feel all the basic investigation should be available at all health care centre, also the health care workers in villages should be given regular training to identify, investigate and referred at earliest at sub district and PHC level for both pulmonary and EPTB because most of the patients are from periphery and referred in late phase of disease to me, [short pause] this can be done by giving money on per patient referral as done in other health program in our country …P12*

#### Category 3.2: Formulation of simple and effective diagnostic protocols for all kinds of TB

All the participants brought up problems with the current diagnostic tools and the complexity of TB diagnosis protocols. They suggested that a TB diagnosis test should be implementable at all peripheral level laboratories. It is necessary for the result to be available the same day, for the test to be easy, cheap and of high sensitivity. They considered that although AFB microscopy is cost-effective and quick, it needs repeated visits to hospital for sampling. They had an opinion that AFB microscopy lacks sensitivity and specificity when performed by technician at the peripheral laboratories. A few participants also felt an extreme need of point-of-care, prognostic or response to treatment markers of TB.

*.....I have a problem regarding the lack of any marker to show the absence of disease in body after a completion of the full course of treatment which in Indian settings is very difficult to conclude or rely on-patients [short pause] I mean if a patient comes after 10 months and says I completed treatment, has no problem right now and asks if I can stop treatment [short pause]now, how I may be sure that he can stop treatment as he is free of the disease in his body now?.P7*

## Discussion

The most important finding of our study is that the confirmation of a diagnosis of TB is done by reviewing the results of multiple tests and diagnostic modalities (radiography, sonography or diagnostic laproscopy), even by experienced and specialist clinicians, to minimize potential misdiagnosis. Most practitioners believe that in real life situations, laboratory confirmation of TB is not possible. Nearly all the laboratory tests for TB, including PCR based tests, are considered non-specific and less sensitive in detecting TB. ‘Empiric treatment’ is preferred over investigation. Though TB diagnosis is free under RNTCP, patients first approach private practitioners with the hope of a quick relief of symptoms, and better facilities. On the other hand, due to the consumerist approach and the pre-set ideas about poor quality of sputum microscopy of doctors, they modify the diagnostic evaluation for patient satisfaction.

The self-reported diagnostic protocols for confirmation of TB are known in India [15] probably because the diagnosis of TB still largely depends on unreliable methods such as smear microscopy, tuberculin test and histology. According to the participants, the diagnosis of all types of TB is still lacking in the areas where it is most prevalent. Furthermore, culture and histology services are not available at any regional or at every district health facility. As about 70 % of the population in low-middle income countries utilizes the peripheral health facility, there is a need to assess the effectiveness of existing tools and to identify alternative point-of-care tests for TB diagnosis with improved accuracy [16, 17].

Several reasons for the doctors ordering a combination of tests and modalities other than culture or PCR were found - (i) Easier process and faster result (ii) Culture or PCR based methods performed outside a public TB program brings about higher expenditure for the patients than even a combination of basic TB investigations (iii) Due to poor sensitivity, the value of culture is questioned (iv) Over-estimation of the reliability of PCR based tests and (v) Ordering PCR for TB investigation is strongly associated with ‘having TB’ by the patient [15].

Our findings elicited that the majority of the doctors come across chronic cough cases in the practice, but very few had explored them. The explanation for the low-rate of ordering of investigations by the doctors is that most symptoms and signs of TB are not specific for diagnosing tuberculosis. Patients with extra-pulmonary TB are even more difficult to diagnose based on symptoms [18]. Previously, many studies have identified drawbacks in the diagnosis of TB patients in routine practice [5–8]. In a study from China, healthcare staff had trouble recognizing TB symptoms, and only severe symptoms such as haemoptysis were likely to get a sputum smear examination [19, 20].

Receiving a TB diagnosis is affected by a dynamic interaction of heterogeneous facts such as provider and patient characteristics, public financing, and health services (general and TB specialist) in rural areas [5, 6, 9]. A comprehensive approach is therefore required to improve diagnosis of TB. A trained healthcare worker is key to fostering a well-functioning TB control program. Our findings suggest that primary care doctors involved in case identification should have sustained support and training for – (i) Awareness of typical and atypical presentations of TB (ii) Early ordering of TB specific tests [21] (iii) Prompt referral of suspected TB case to higher centers. Referrals can be enhanced through an incentive mechanism. Participants believe that TB investigation promotion campaigns for private and public healthcare providers in the form of education for- screening for TB in high-risk groups, sputum smear procedure and result interpretation, may increase awareness and usage of basic and newer TB diagnostics. Mobile laboratory services may ensure access to services for potential TB patients in rural and poor areas. The National TB program needs to strengthen the laboratories at local level and provide basic functioning equipment. There is also a need to demonstrate the impact of diagnostic-interventions in patient-relevant outcomes, and acceptability by the clinicians [22].

Our findings need to be interpreted in the light of few limitations. The possibility of social desirability cannot be ruled out, as the interviewer (first author) is a known pathologist to the interviewees. Practitioners might have given what they considered desirable answers that reflect their knowledge rather than the actual practice of the mentioned diagnostic method. A reluctance to disclose the practice of prescribing outdated tests, or false statements about ordering newer tests, may have resulted in findings that doctors practice according to recommendations [23]. However, persistent observation by the first author of the interviewee’s culture and practice habits helped in improving the credibility of the data [24]. The participants had good rapport and were not hesitant to the interviewer, as nearly all (16/18) agreed to participate in study when approached. The trustworthiness of our findings was also ensured through interviewing health professionals of a wide variety of specialties, age, experience and from both public and private sectors, and by the independent agreement about the data’s meaning by the authors [25].

We note that in regions of the world which are resource-poor and have a high TB burden, it is important that clinicians use an adequate clinical screening approach to identify patients suspected to have TB, otherwise laboratory services will be overburdened with unnecessary testing. However simultaneously, it is important to realize that at present there is no stand-alone test for the rapid detection of tuberculosis in all patients in resource-limited settings. TB diagnostic tests should be carefully incorporated in country’s national TB diagnostic algorithm for realistic future use. The inefficient laboratory confirmation of TB may indirectly lead to an increase in the total cost burden of multiple tests to the patient, and the empiric treatment may increase the possibility of the development of multi-drug resistant TB.

## Conclusions

The medical specialist use ‘multiple-tests’ and ‘empiric-treatment’ as diagnostic tests with low dependence and uptake of offered laboratory TB investigations. Consequently, building up the capacity and enhancing universal access to rapid and accurate laboratory diagnostics are necessary to control TB in resource limited endemic countries.
